# *Acanthamoeba castellanii* cysteine protease 3 promotes M1 macrophage polarization through the TLR4/NF‑κB pathway

**DOI:** 10.1186/s13071-025-07060-y

**Published:** 2025-10-29

**Authors:** Zhi-xin Wang, Wan-jun Jiao, Mian-jing Wang, Yong Yang, Hai-long Wang, Hong-li Liu

**Affiliations:** 1 School of Basic Medicine, Basic Medical Sciences Center, Shanxi Medical University, Jinzhong, 030600 Shanxi China; 2 Shanxi Key Laboratory of Functional Proteins, School of Basic Medical Science, Shanxi Medical University, Taiyuan, 030001 Shanxi China

**Keywords:** *Acanthamoeba castellanii*, Cysteine protease 3, Macrophage polarization, TLR4/NF-κB pathway, Inflammation

## Abstract

**Background:**

*Acanthamoeba* spp., which are free-living protozoan parasites, are etiological agents for *Acanthamoeba* keratitis and granulomatous amoebic encephalitis. Macrophages participate in the host defense response to resist *Acanthamoeba* spp. This study examined the effect of *Acanthamoeba castellanii* cysteine protease 3 (*Ac*CP3) on macrophage activation during inflammatory responses and explored the underlying mechanisms.

**Methods:**

The effects of recombinant *Ac*CP3 (r*Ac*-CP3) stimulation on inflammatory factor levels and macrophage polarization were examined using murine macrophage cells (RAW264.7 cells). Western blotting assay was carried out for analyzing TLR4/NF‑κB pathway-related protein levels. In addition, phosphorylated NF-κB was examined for its nuclear transport using immunofluorescence. The effect of the NF-κB inhibitor pyrrolidinedithiocarbamate ammonium (PDTC) on r*Ac*-CP3-induced M1 polarization was analyzed. Furthermore, RAW264.7 cells were co-cultivated using *Ac*CP3 knockdown trophozoites to examine indicators of M1 polarization and pathway-related protein levels.

**Results:**

As revealed by quantitative real-time polymerase chain reaction (qRT-PCR), western blotting, and enzyme-linked immunosorbent assays, treatment with r*Ac*-CP3 upregulated the mRNA, protein, and secretion levels, respectively, of Il6, Il1b, Tnfa, and Ifng in macrophages. Flow cytometric analysis demonstrated that r*Ac*-CP3 promoted Cd86^+^ cell (macrophage) proliferation. Additionally, r*Ac*-CP3 upregulated Nos2 expression and nitric oxide (NO) production, indicating that r*Ac*-CP3 promotes macrophage polarization toward an M1-like phenotype. Kyoto Encyclopedia of Genes and Genomes pathway enrichment analysis demonstrated that the NF-κB pathway was among the top 20 significantly enriched pathways. Treatment with r*Ac*-CP3 upregulated the levels of Tlr4, p-Rela, and p-Nfkbia in RAW264.7 cells. Immunofluorescence analysis demonstrated the nuclear translocation of p-Rela. Pretreatment with the NF-κB inhibitor PDTC downregulated the Tlr4, p-Rela, and p-Nfkbia levels in r*Ac*-CP3-treated cells. Additionally, PDTC significantly mitigated the r*Ac*-CP3-induced upregulation of Nos2, NO and pro-inflammatory factor production. *Ac*CP3 knockdown decreased the number of Cd86^+^ cells and suppressed *Acanthamoeba* trophozoite-induced Nos2 upregulation and NO production. Additionally, *Ac*CP3 knockdown downregulated Tlr4, p-Rela, and p-Nfkbia in RAW264.7 cells. PDTC and *Ac*CP3 knockdown suppressed the r*Ac*-CP3-induced M1 macrophage polarization.

**Conclusions:**

*Ac*CP3 promotes M1 macrophage polarization through the TLR4/NF-κB pathway and may exacerbate inflammation through upregulating pro-inflammatory cytokines.

**Graphical Abstract:**

**Figure:**
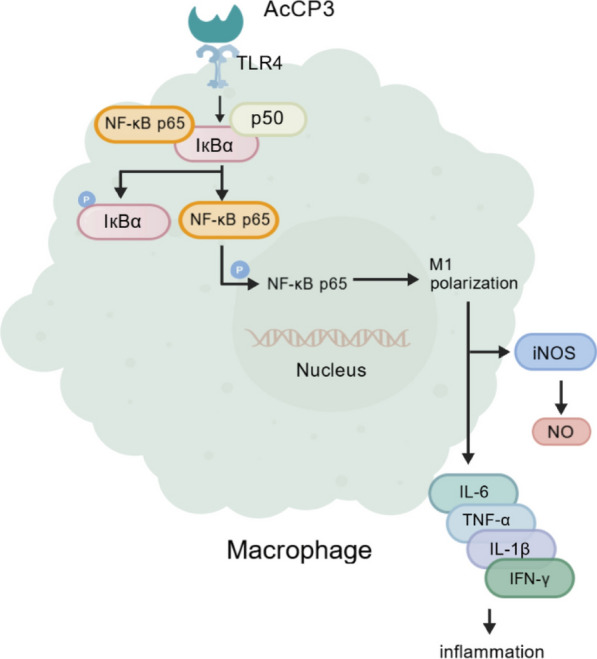

**Supplementary Information:**

The online version contains supplementary material available at 10.1186/s13071-025-07060-y.

## Background

*Acanthamoeba castellanii*, a free-living protozoan that is widely distributed in nature, is the etiological agent for granulomatous amoebic encephalitis and *Acanthamoeba* keratitis (AK). AK, a severe corneal infection, can lead to irreversible visual sequelae. Treating AK is challenging as it is diagnosed at advanced stages, resistant to standard therapies, and a chronic condition. The annual incidence of AK is increasing, especially among contact lens users. AK is associated with corneal trauma, such as that caused by sewage exposure [1, 2]. Thus, the treatment of AK is challenging.

Macrophages, an innate immune system component, are involved in immune responses against pathological conditions. Previous studies have reported that macrophages are commonly detected in AK biopsies [3]. Macrophages are involved in the immune responses against *Acanthamoeba* spp., promoting protozoan clearance [3–5]. During the early stages of ocular infections, the subconjunctival injection of liposomes containing clodronate (a macrophage-depleting drug) exacerbates AK symptoms in Chinese hamsters [6]. Anti-*Acanthamoeba* antibody and interferon gamma (IFN-γ) significantly potentiate the trophozoite clearance capacity of macrophages in vitro, indicating the role of activated macrophages in trophozoite clearance [7]. Additionally, macrophages can initiate and maintain effective immune responses and promote tissue repair [8, 9]. *Acanthamoeba* induces the activation of macrophages and the production of interleukin (IL)-6 and IL-12 through myeloid differentiation primary response 88 (MyD88)-related and Toll-like receptor (TLR) 4-related mechanisms [8]. Macrophages are highly plastic, exhibiting diverse phenotypes and functions. In particular, macrophages undergo classically activated (M1) and alternatively activated (M2) polarization [10]. IFN-γ and lipopolysaccharide (LPS) alone or in combination promote the M1 polarization through nuclear factor kappa-light-chain-enhancer of activated B cells (NF-κB) and signal transducer and activator of transcription 1 (STAT1) transcription factors. Inducible nitric oxide synthase (iNOS) and pro-inflammatory cytokines (like IL-1β, IL-6, tumor necrosis factor α (TNFα)) expressed in pro-inflammatory cells initiate inflammatory responses for pathogen clearance [11, 12].

Cysteine proteases (CPs), which are multifunctional enzymes, regulate the life cycle of some protozoans through the modulation of multiple processes, including nutrient intake, parasite protein degradation, host immune response evasion or modulation, host cell/tissue invasion, and pathogenicity. *Acanthamoeba* CPs are reported to exert cytotoxic effects against host cells [13]. Additionally, *Acanthamoeba* CPs hydrolyze proteins, including albumin, fibronectin, hemoglobin, immunoglobulin A (IgA), and immunoglobulin G (IgG) [14, 15]. Cyst-specific CP with papain and cathepsin B-like activity is essential for the mitochondrial autophagy stage during cyst formation of *A. castellanii* [13]. Previously, we reported that CP6 from *A. castellanii* (*Ac*CP6) was correlated with encystation and that *A. castellanii* CP3 (*Ac*CP3) is an important virulence protein with hydrolytic activity against host hemoglobin, collagen, and albumin, which may activate the Ras/Raf/Erk/p53 signaling pathway in corneal epithelial cells, leading to lesion development [16]. The mechanisms through which *Ac*CP3 induces macrophage polarization and regulates the immune microenvironment remain to be further explored.

The present work hypothesized that *Ac*CP3 induces M1 macrophage polarization. Mouse macrophages (RAW264.7 cells) were stimulated with recombinant *Ac*-CP3 (r*Ac*-CP3) or *Ac*CP3 knockdown *Acanthamoeba* trophozoites. Treatment with r*Ac*-CP3 promoted M1 macrophage polarization and pro-inflammatory factor production. This study also examined the M1 polarization-related pathways. *Ac*CP3 enhanced the differentiation of M0 macrophages into M1 macrophages via the TLR4/NF-κB pathway.

## Methods

### Amoeba and cell culture

*A. castellanii* provided by the American Type Culture Collection (ATCC 30011) was subjected to axenic culture using peptone–yeast–glucose medium under 26 °C. The cultured *A. castellanii* protozoa were seeded in 75-cm^2^ flasks and incubated until a 90% density of trophozoites was achieved. RAW264.7 cells were provided by ATCC and cultivated within Dulbecco’s modified Eagle’s medium (DMEM) (Gibco, USA) that contained 10% fetal bovine serum (FBS) (Thermo Fisher, USA) as well as 1% penicillin–streptomycin (Gibco, USA). Human corneal epithelial cells (HCECs) obtained from Bioleaf (Shanghai, China) were cultivated within DMEM (Gibco, USA) that contained 10% FBS as well as 1% penicillin–streptomycin. All cells underwent culture under 37 °C with 5% CO_2_.

### Expression, purification, and refolding of r*Ac*-CP3

r*Ac*-CP3 was expressed, purified, and refolded as described previously [16]. The size and purity of r*Ac*-CP3 were analyzed using sodium dodecyl sulfate–polyacrylamide gel electrophoresis (SDS-PAGE). Meanwhile, protein concentration was analyzed with Pierce bicinchoninic acid protein assay kit (Thermo Fisher Scientific, USA). The endotoxin level in the r*Ac*-CP3 sample was examined using the Toxin Sensor^TM^ gel clot endotoxin assay kit (Genscript, China). The levels of endotoxin in r*Ac*-CP3 were within the physiological range, conforming to the national standard of the People’s Republic of China for medical products. Thus, the r*Ac*-CP3 could be used for subsequent experiments.

### Cell viability test

Logarithmic-phase RAW264.7 cells were inoculated into 24-well plates before an additional 24 and 48 h of incubation using r*Ac*-CP3 at varying doses (5, 10, 20, 30, and 50 μg/mL). Next, these cells underwent incubation using the cell counting kit-8 (CCK-8) solution for 0.5–2 h to determine cell viability. The absorbance of the reaction mixture at 450 nm was measured with the microplate reader (SpectraMax iD3 & iD5, USA). Cell viability assay was independently repeated three times.

### In vitro stimulation of RAW264.7 cells with r*Ac*-CP3

RAW264.7 cells were allowed to adhere to the wells of 24-well plates overnight and stimulated with specified doses of r*Ac*-CP3 for 24 and 48 h. Cells treated with Tris–HCl (20 mM, pH 8.0) and LPS (1 μg/mL) served as the negative and positive controls, separately. Total cellular RNA (ribonucleic acid) and proteins were isolated to perform quantitative real-time polymerase chain reaction (qRT-PCR), RNA sequencing, western blotting, and flow cytometry assays. The NO and IL-1β levels in RAW264.7 cells were analyzed by the Griess assay and enzyme-linked immunosorbent assay (ELISA), respectively.

### qRT-PCR analysis

An RNeasy^®^ Plus Mini kit (Qiagen, Germany) was utilized to isolate total RNA, which was later subjected to reverse-transcription in complementary DNA (cDNA) with PrimeScript™ RT reagent kit (Takara, Japan). cDNA later underwent qRT-PCR analysis with TB Green Premix Ex Taq II (Takara, Japan). All primers used in qRT-PCR analysis can be obtained from Additional file 1: Table S1. *Mus-actin* served as a housekeeping gene. The relative gene level was determined by the 2^−ΔΔCt^ approach. Every sample was examined thrice.

### Western blotting analysis

Adherent RAW264.7 cells (10^6^ cells/well) within six-well plates received 24 h of incubation using 10 µg/mL r*Ac*-CP3 or Tris–HCl (20 mM, pH 8.0). The lysates of treated cells were subjected to western blotting. Briefly, RAW264.7 cells were harvested using 10% phosphate-buffered saline (PBS; pH 7.4) that contained protease inhibitor cocktail (MedChemExpress, USA) and phosphatase inhibitor cocktail 2 (Boster, China). Total protein underwent SDS-PAGE with the 10% gel. Thereafter, those proteins resolved were added into the polyvinylidene fluoride membrane. After 1 h of blocking using 5% milk or 5% bovine serum albumin (BSA) under ambient temperature, the membrane further underwent overnight incubation using primary antibodies against IFN-γ (Wanleibio, China), IL-1β (Wanleibio, China), iNOS (Abways, China), TLR4 (ABclonal, China), NF-κB p65 (CST, USA), phospho-NF-κB p65 (Abways, China), IκBα (CST, USA), phospho–IκBα (Ser32) (CST, USA), β-actin (Proteintech, China), GAPDH (Bioss, China), and tubulin (HUABIO, China) under 4 °C, and later further incubation using horseradish peroxidase-conjugated goat anti-rabbit or anti-mouse IgG (Abcam, UK) under ambient temperature. Immunoreactive signals were visualized with the ChemiDoc MP Imaging System (Bio-Rad, USA) and quantified using ImageJ software.

### ELISA

The culture supernatant of RAW264.7 cells incubated with r*Ac*-CP3 for 24 h was harvested to examine the Il1b level using the mouse IL-1 beta ELISA kit (Thermo Fisher Scientific, USA). This assay was repeated three times.

### Griess assay

The culture supernatant of RAW264.7 cells after 24 h of r*Ac-*CP3 incubation was used to determine the levels of nitrites and nitrates using fluorometry NO assay kit (Beyotime Biotechnology Inc, China). The NO levels were normalized to the protein content. This assay was performed thrice.

### Flow cytometry

RAW264.7 cells after 24 h of r*Ac*-CP3 incubation were incubated with phycoerythrin-conjugated anti-mouse CD86 antibody (Elabscience, China) for a 30-min duration under 4 °C. The cells were rinsed, resuspended, and examined using the FACSVelsta™ flow cytometer (BD Biosciences).

### RNA sequencing and differential analysis

Total RNA was isolated in RAW264.7 cells after 24 h of r*Ac*-CP3 incubation using an RNeasy Plus Mini kit. At least three biological replicates were used for every sample. High-quality total RNA (1 μg) was applied in preparing the RNA-seq transcriptome library based on Illumina^®^ Stranded mRNA Prep Ligation instructions (Illumina). Raw paired-end reads later underwent trimming and quality control with fastp using default parameters. HISAT2 software was utilized to align clean reads with reference genome in the orientation mode. StringTie was used to assemble mapped reads in experimental and control groups using the reference-based method. Differentially expressed genes (DEGs) in two groups were analyzed by DESeq2 or DEGseq. Functional enrichment analysis of DEGs was performed with Goatools and KOBAS.

### Immunofluorescence analysis

RAW264.7 cells were inoculated onto sterile glass coverslips and seeded into 24-well plates (5 × 10^4^/well). After the cells reached the specific density, they were incubated with r*Ac*-CP3 for 2 h. RAW264.7 cells on coverslips were immersed within 4% formaldehyde for 15 min, followed by 15 min of permeabilization using 0.2% Triton X‐100 in PBS, and 1 h of blocking using 5% BSA. Next, RAW264.7 cells received incubation using anti-phospho-NF-κB p65 (CST, USA) primary antibodies at ambient temperature, followed by incubation with Alexa Fluor 488-conjugated goat anti-rabbit IgG secondary antibody (Thermo Fisher, USA) at ambient temperature in the dark. After washing, the nuclei of cells were stained with 4′,6-diamidino-2-phenylindole (BOSTER, China) to visualize the nuclei. The samples were mounted, sealed, and monitored with the confocal microscope. Confocal images of cell nuclei were captured using an FV3000 confocal microscope (Olympus, Japan).

### Evaluation of pyrrolidinedithiocarbamate ammonium (PDTC) effects

RAW264.7 cells cultured within six-well plates received 1 h of incubation using 50 μM PDTC (NF-κB inhibitor; Beyotime, China) [17] and later incubation using r*Ac*-CP3 (10 μg/mL) for evaluating the role of PDTC in NF-κB pathway. Cells treated with Tris–HCl, PDTC, and r*Ac*-CP3 served as controls. The TLR4/NF-κB pathway-related protein levels were quantified through western blotting assay. Meanwhile, Nos2 mRNA and protein levels were analyzed by qRT-PCR and western blotting assays separately. The NO content within culture supernatant was quantified using the Griess assay.

### Evaluation of the effect of *Ac*CP3 knockdown trophozoites on *Acanthamoeba*‑mediated macrophage M1 polarization

*Ac*CP3 was knocked down as described previously using small interfering RNA (siRNA) [16]. RAW264.7 cells within six-well plates were subjected to 12 h of incubation using trophozoites under 37 °C and 5% CO_2_ conditions. *Ac*CP3 knockdown trophozoites were co-cultured with RAW264.7 cells (multiplicity of infection, 1:200). The control groups comprised RAW264.7 cells co-cultured with wild-type trophozoites or negative control siRNA-transfected trophozoites and routinely cultured RAW264.7 cells. The culture supernatant was harvested to determine the NO content using the Griess reagent. The trophozoites were discarded, and the RAW264.7 cells were collected. The Nos2 level was quantified using western blotting analysis. CD86-labeled macrophages were identified using flow cytometry. Then, the TLR4/NF-κB pathway-related protein levels were quantified by western blotting.

### Statistical analysis

GraphPad Prism 8 (GraphPad Software, USA) was adopted for statistical analysis and graphing. The results are presented by mean ± standard deviation from triplicate assays. Means in two groups were compared by Student’s *t*-test or one-way and two-way analysis of variance. After standardizing the *z*-score among diverse rows, hierarchical clustering was performed. Heatmaps were not grouped. Differences were considered significant at *P* < 0.05 (ns, not significant; ^*^P < 0.05, ^**^P < 0.01, ^***^P < 0.001, and ^****^P < 0.0001).

## Results

### *Ac*CP3 upregulates the production of pro-inflammatory factors

*Acanthamoeba* trophozoites were co-cultured with HCECs. qRT-PCR assay (Additional file 1: Fig. S1A, B) revealed that the *IL6*, *IL8*, and *IL1B* mRNA levels in HCECs co-cultured with trophozoites apparently increased relative to control HCECs. Meanwhile, *AcCP3* mRNA expression in trophozoites co-cultured with HCECs was significantly higher than that in controls. As shown in Supplementary Fig. S1C, r*Ac*-CP3 significantly upregulated the *IL6* and *IL8* mRNA levels in HCECs. These findings suggest that *Ac*CP3 enhances pro-inflammatory cytokine secretion in HCECs.

In the cornea, macrophages are involved in intrinsic immune responses [18], especially against infection [19]. Thus, this study selected macrophages for further study.

The viability of RAW264.7 cells treated with r*Ac*-CP3 or Tris–HCl for 24 and 48 h at indicated doses was examined using the CCK-8 assay. Compared with that of control cells, the viability of RAW264.7 cells was not significantly affected upon treatment with 5–10 μg/mL r*Ac-*CP3 at 24 and 48 h and 20 μg/mL r*Ac-*CP3 at 24 h, while the viability of cells markedly increased upon treatment with 30–50 μg/mL r*Ac*-CP3 at 24 and 48 h and 20 μg/mL r*Ac-*CP3 at 48 h (Additional file 1: Fig. S2).

**Fig. 1:**
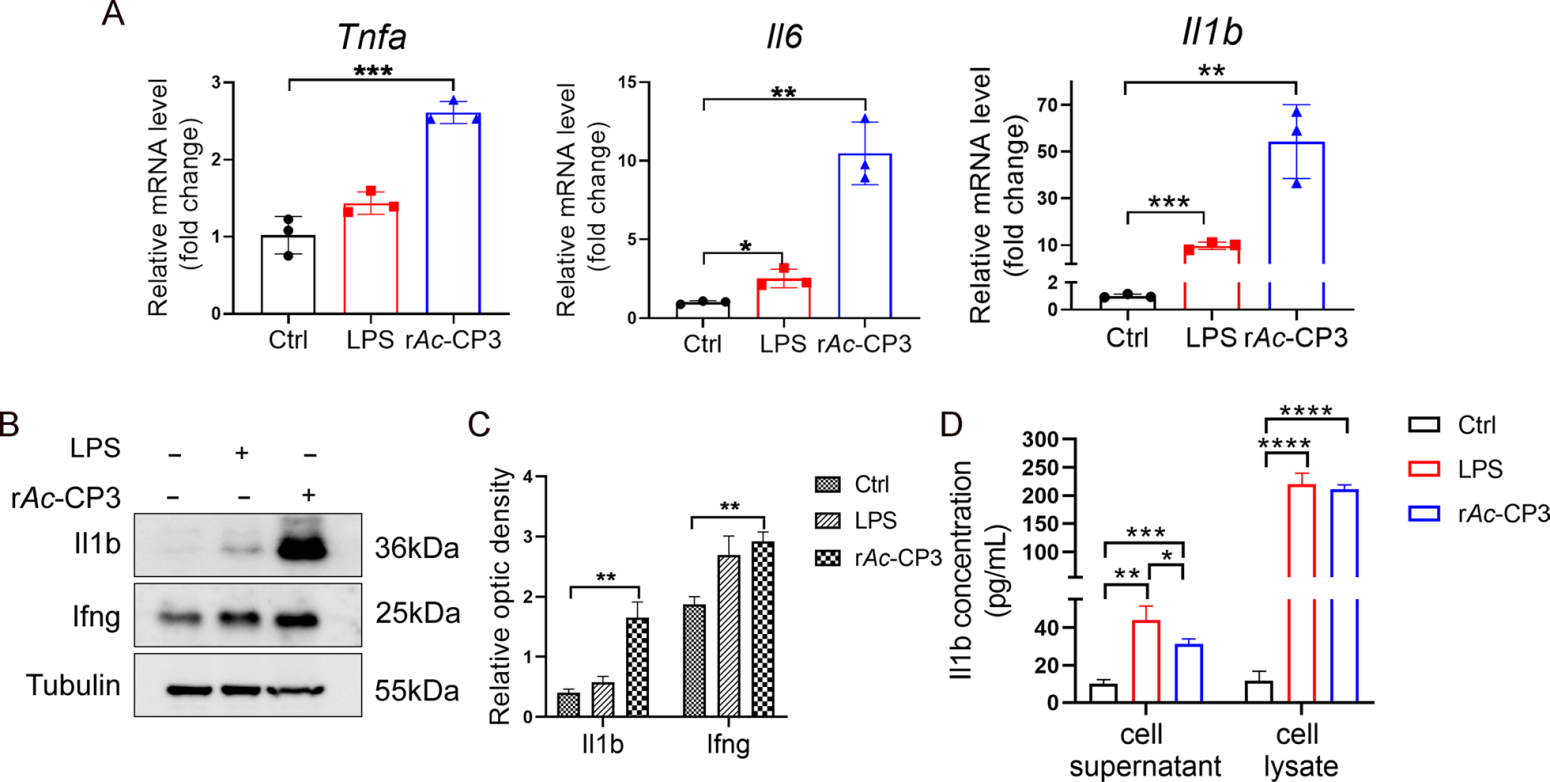
Recombinant *A. castellanii* cysteine protease 3 (r*Ac-*CP3) promotes pro-inflammatory cytokine production. RAW264.7 cells underwent 24 h of r*Ac*-CP3 or LPS treatment. Cells that received Tris-HCl treatment served as the control. **A** The mRNA levels of the pro-inflammatory cytokines *Tnfa*, *Il6*, and *Il1b* were analyzed within RAW264.7 cells by qRT-PCR assay. **B** The Il1b and Ifng levels within RAW264.7 cells after r*Ac*-CP3 or LPS treatment were examined by western blotting analysis. **C** The optical densities of Il1b and Ifng within RAW264.7 cells were analyzed using ImageJ. **D** The Il1b levels in the culture supernatant and cell lysates were measured by ELISA

Next, the function of r*Ac*-CP3 in pro-inflammatory factors was examined within RAW264.7 cells using qRT-PCR, western blotting, and ELISA analyses. Treatment with r*Ac*-CP3 significantly upregulated the *Tnfa*, *Il6*, and *Il1b* mRNA levels (Fig. 1A). Additionally, r*Ac*-CP3 markedly upregulated Il1b and Ifng levels (Fig. 1B–D). Furthermore, the secretion of Il1b was upregulated in the culture supernatant of r*Ac-*CP3-treated cells (Fig. 1D). These results suggest that *Ac*CP3 effectively promotes pro-inflammatory cytokine secretion within RAW264.7 cells.

### r*Ac*-CP3 promotes M1 polarization in macrophages

**Fig. 2:**
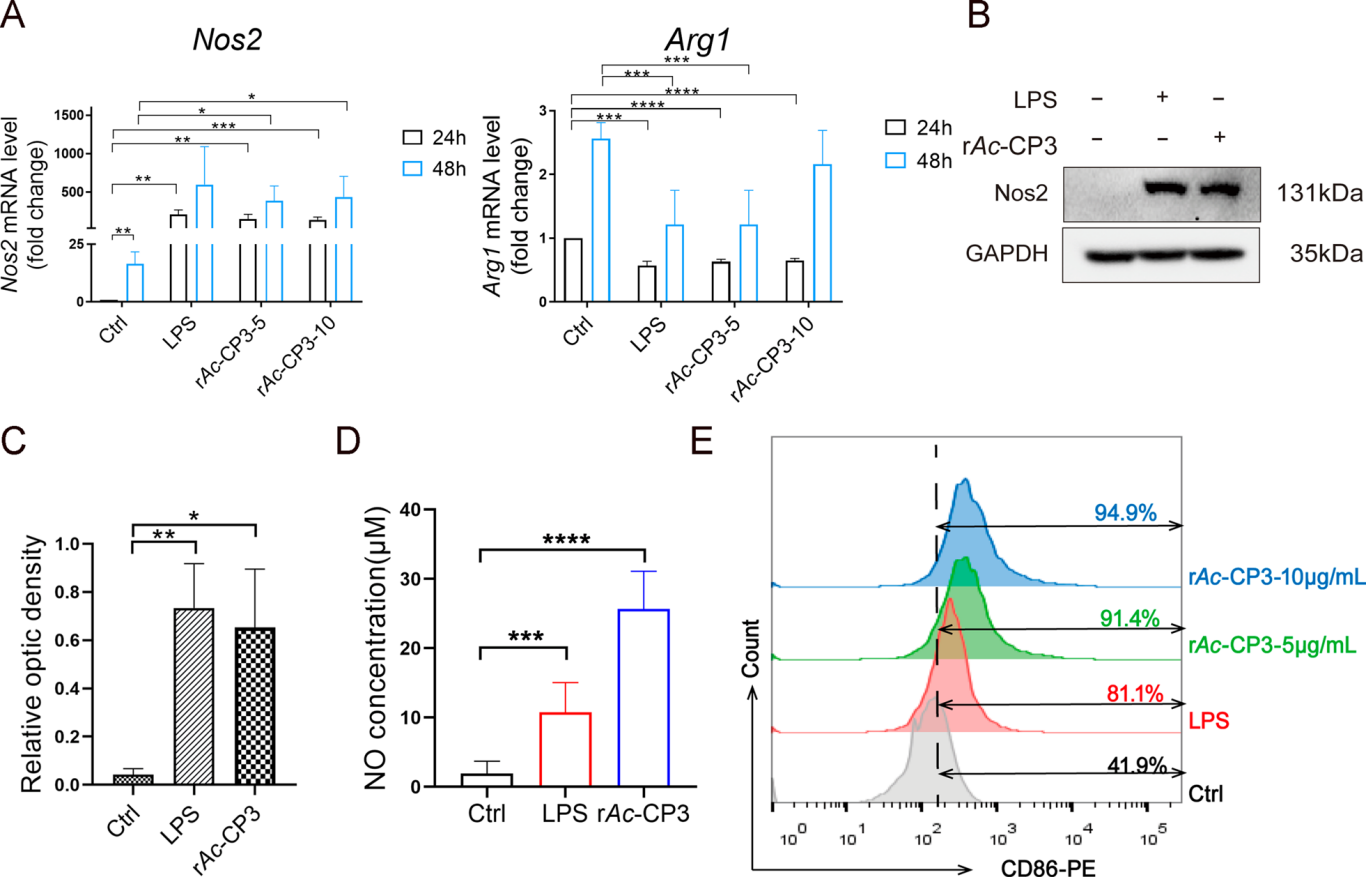
Recombinant *A. castellanii* cysteine protease 3 (r*Ac*-CP3) promoted M1 polarization. **A** The mRNA levels of macrophage polarization-related markers in RAW264.7 cells after r*Ac*-CP3 or LPS treatment were examined by qRT-PCR.** B** Nos2 levels within RAW264.7 cells after r*Ac*-CP3 or LPS treatment were analyzed by western blotting. **C** The Nos2 optical densities of RAW264.7 cells.** D** Nitric oxide (NO) levels within in the supernatant of RAW264.7 cells after r*Ac*-CP3 or LPS treatment were quantified using the Griess assay. **E** RAW264.7 cells after 24 h of r*Ac*-CP3 or LPS treatment were subjected to Cd86 (an M1 macrophage marker) immunostaining and flow cytometry.

This study examined markers of macrophage polarization in RAW264.7 cells after r*Ac*-CP3 or LPS treatment for 24 and 48 h. iNOS expression is an identification marker for M1 macrophages. As suggested by qRT-PCR analysis, *Nos2* mRNA expression of r*Ac*-CP3-treated group increased relative to control group at 24 and 48 h (Fig. 2A). In contrast, the mRNA levels of *Arg1*, an M2 polarization marker, were downregulated in the r*Ac*-CP3-treated group. From western blotting analysis, Nos2 levels expression in LPS-treated and r*Ac*-CP3-treated groups increased (Fig. 2B, C). These findings indicate that r*Ac*-CP3 upregulates the mRNA and protein levels of the M1 marker Nos2 in RAW264.7 cells. iNOS catalyzes the reaction with arginine to generate NO to kill invading pathogens. This study analyzed the NO level in the culture supernatant of r*Ac*-CP3-treated or LPS-treated cells using the Griess assay. As shown in Fig. 2D, r*Ac*-CP3 upregulated the NO levels. CD86 is a marker for M1 macrophages. As shown in Fig. 2E, the percentage of Cd86^+^ macrophages in the LPS-treated and r*Ac*-CP3-treated groups increased compared with control group. Consequently, *Ac*CP3 promotes M1 polarization of RAW264.7 cells.

### r*Ac*-CP3 enhances M0 macrophage differentiation into M1 macrophages via TLR4/NF-κB pathway

**Fig. 3:**
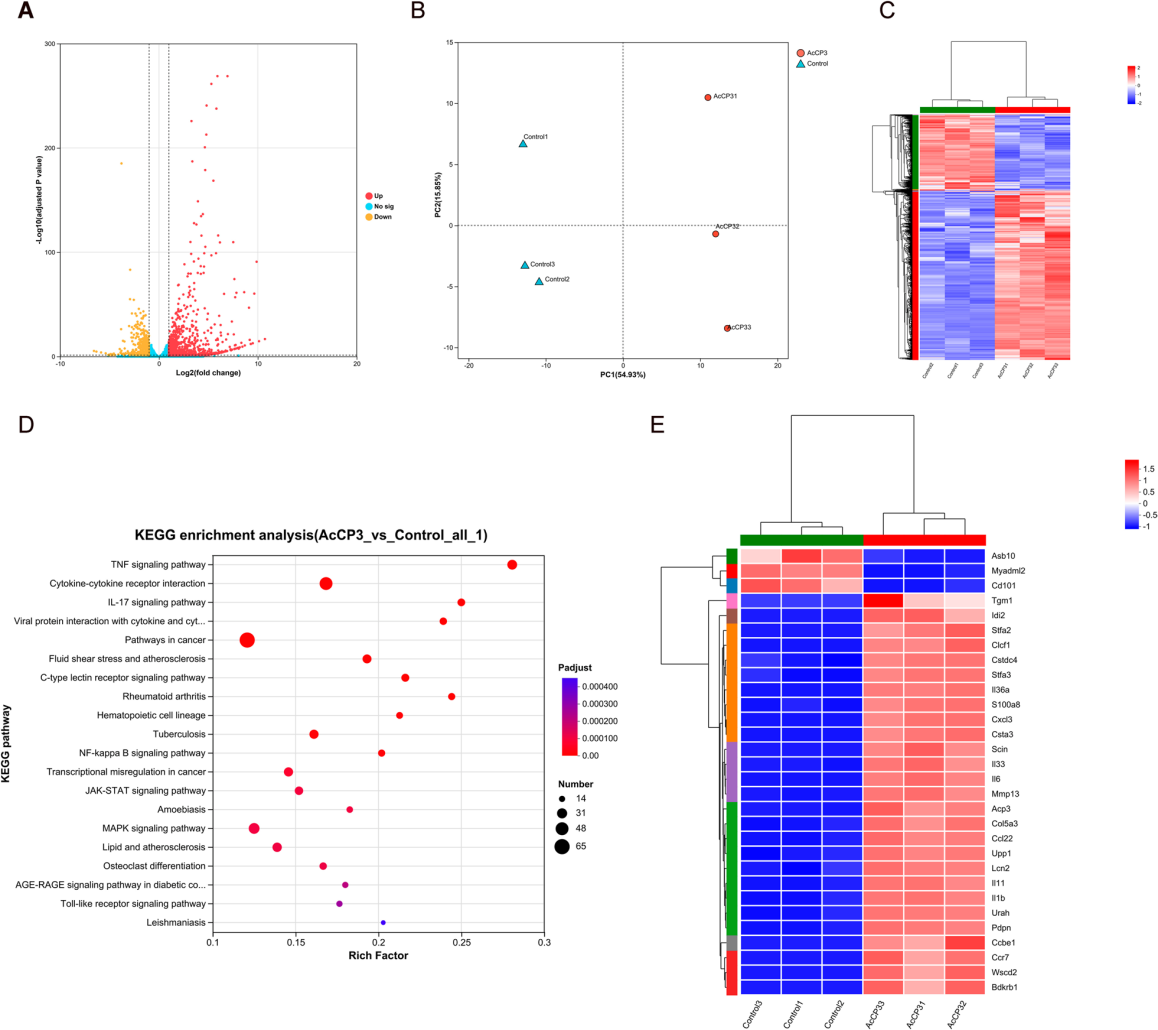
Effect of recombinant *A. castellanii* cysteine protease 3 (r*Ac*-CP3) on the transcriptome of RAW264.7 cells. **A** The volcano plot exhibiting DEGs between r*Ac*-CP3-treated and control RAW264.7 cells. **B** Principal component analysis of genes in r*Ac*-CP3-treated RAW264.7 cells. **C** Heatmap for DEGs in r*Ac*-CP3-treated RAW264.7 cells. **D** The top 20 KEGG pathways enriched by DEGs between r*Ac*-CP3-treated and control RAW264.7 cells. **E** Heatmap for 30 most significant DEGs between the control and r*Ac*-CP3-treated groups

This study examined effects of r*Ac*-CP3 on the transcriptome of macrophages. In particular, r*Ac*-CP3-treated RAW264.7 cells were subjected to whole-transcriptome RNA sequencing. As shown in Fig. 3A, 1605 DEGs were identified between the r*Ac*-CP3-treated and control groups (496 downregulated genes and 1109 upregulated genes). Multivariate regression (principal component analysis) and hierarchical clustering analyses revealed that the diversity of genes in the r*Ac*-CP3-treated group increased compared with control group (Fig. 3B, C). Figure 3E shows the 30 most significant DEGs. Some cytokines (*Il6* and *Il1b*) and chemokines (*Ccl22* and *Cxcl3*) were significantly upregulated. Kyoto Encyclopedia of Genes and Genomes (KEGG) analysis revealed that the NF-κB pathway was among the top 20 enriched pathways (Fig. 3D). The NF-κB pathway is reported to regulate inflammation. NF-κB and STAT1 are the main transcription factors of M1 macrophages [20]. Thus, r*Ac*-CP3 may regulate inflammatory responses of M1 macrophages through the NF-κB pathway.

**Fig. 4:**
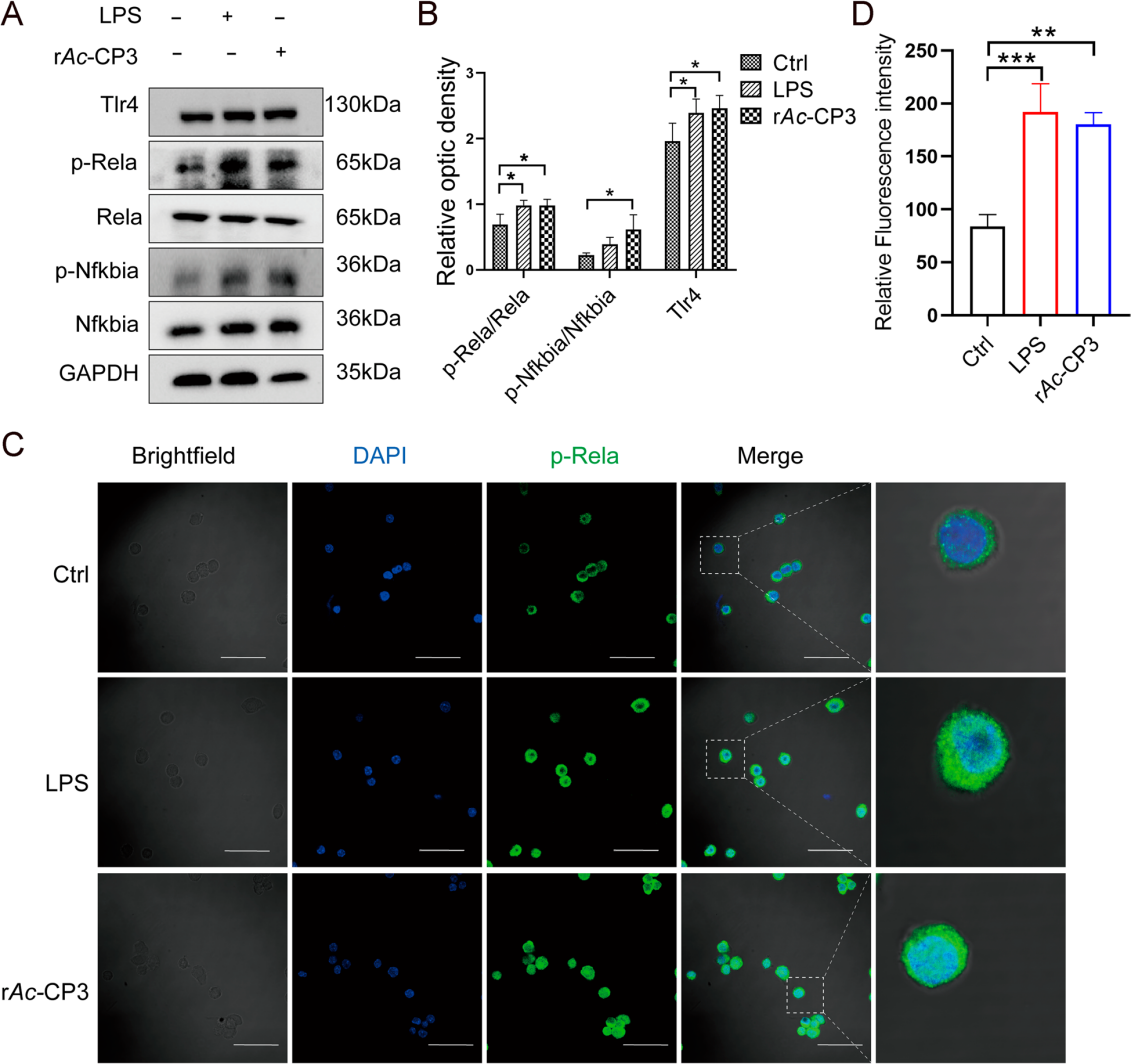
Recombinant *A. castellanii* cysteine protease 3 (r*Ac*-CP3) activates TLR4/NF-κB pathway. **A** Rela, p-Rela, Nfkbia, p-Nfkbia, and Tlr4 levels in RAW264.7 cells after 24 h of r*Ac*-CP3 or LPS treatment were analyzed by western blotting analysis. **B** The graph quantifying protein band intensities. **C** The levels of p-Rela in cells after 2 h of r*Ac*-CP3 treatment were examined using immunofluorescence staining. 4′,6-Diamidino-2-phenylindole (DAPI) was added to for nuclear staining (blue). Scale bar: 50 μm. **D** The quantification of nuclear p-Rela fluorescence intensity

Next, this study examined the effect of the NF‑κB pathway on r*Ac*-CP3-induced M1 polarization. From western blotting assay, r*Ac*-CP3 increased p-Nfkbia and p-Rela levels in RAW264.7 cells (Fig. 4A, B). Similarly, r*Ac*-CP3 upregulated the Tlr4 level in RAW264.7 cells. Furthermore, immunofluorescence analysis revealed a distinct green fluorescence signal within cell nuclei after r*Ac*-CP3 treatment (Fig. 4C, D), indicating nuclear translocation of p-Rela. From the above results, r*Ac*-CP3 upregulates TLR4/NF‑κB pathway-related protein levels and the nuclear translocation of p-Rela in RAW264.7 cells. Thus, r*Ac*-CP3 enhances M1 macrophage polarization and pro-inflammatory factor secretion through the TLR4/NF-κB pathway.

### PDTC mitigates r*Ac*-CP3-induced M1 polarization

**Fig. 5:**
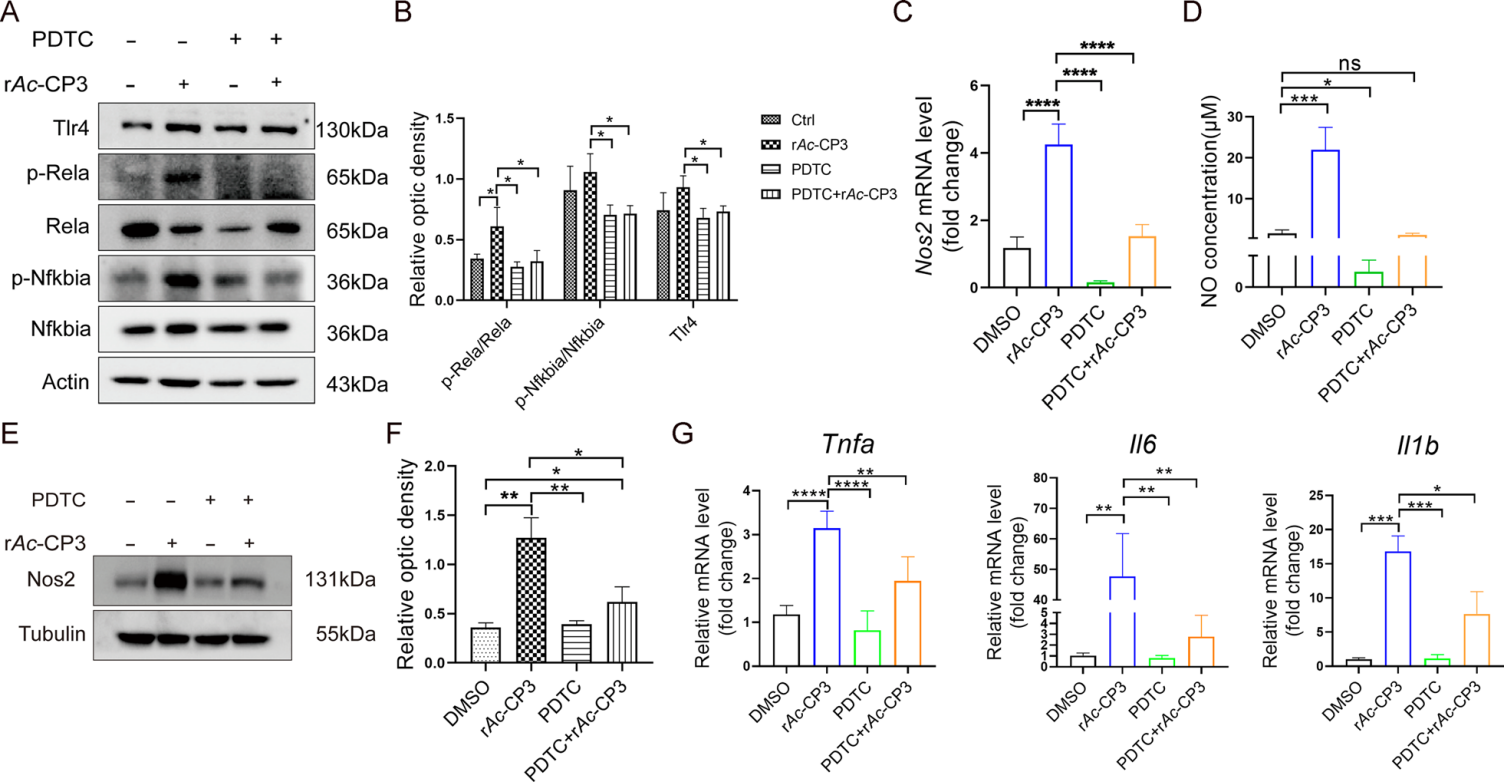
Pyrrolidinedithiocarbamate ammonium (PDTC) pretreatment suppresses recombinant *A. castellanii* cysteine protease 3 (r*Ac*-CP3)-induced M1 polarization via TLR4/NF‑κB pathway. RAW264.7 cells underwent 24 h of r*Ac*-CP3, PDTC, or r*Ac*-CP3 + PDTC treatment. **A** Rela, p-Rela, Nfkbia, p-Nfkbia, and Tlr4 levels were analyzed by western blotting analysis. **B** The graphs quantifying protein band intensities. **C** The *Nos2* mRNA level was analyzed by qRT-PCR assay. **D** Quantification of NO in culture supernatant using the Griess assay. **E** The Nos2 level was analyzed by western blotting. **F** The graphs showing the quantification of protein band intensities. **G** *Tnfa*, *Il6*, and *Il1b* mRNA levels within RAW264.7 cells were analyzed by qRT-PCR assay

To examine whether r*Ac*-CP3 promotes M1 macrophage polarization by activating the TLR4/NF‑κB pathway, the levels of TLR4/NF-κB pathway-related proteins were determined in r*Ac*-CP3-treated RAW264.7 cells in the presence or absence of PDTC. Western blotting analyses revealed that PDTC pretreatment mitigated the r*Ac*-CP3-induced upregulation of Tlr4, p-Rela, and p-Nfkbia levels (Fig. 5A, B). Similarly, PDTC pretreatment suppressed the r*Ac*-CP3-induced upregulation of Nos2 in RAW264.7 cells (Fig. 5C, E, F). As shown in Fig. 5D, PDTC downregulated NO production in r*Ac*-CP3-treated cells. From Fig. 5G, *Tnfa*, *Il6*, and *Il1b* mRNA levels were downregulated in PDTC-treated macrophages. PDTC suppressed pro-inflammatory factor generation. Thus, *Ac*CP3 may downregulate pro-inflammatory factors through the NF-κB pathway. These results demonstrated that PDTC mitigates r*Ac*-CP3-induced M1 polarization via TLR4/NF‑κB pathway.

### *Ac*CP3 knockdown suppresses M1 polarization through TLR4/NF-κB pathway

**Fig. 6:**
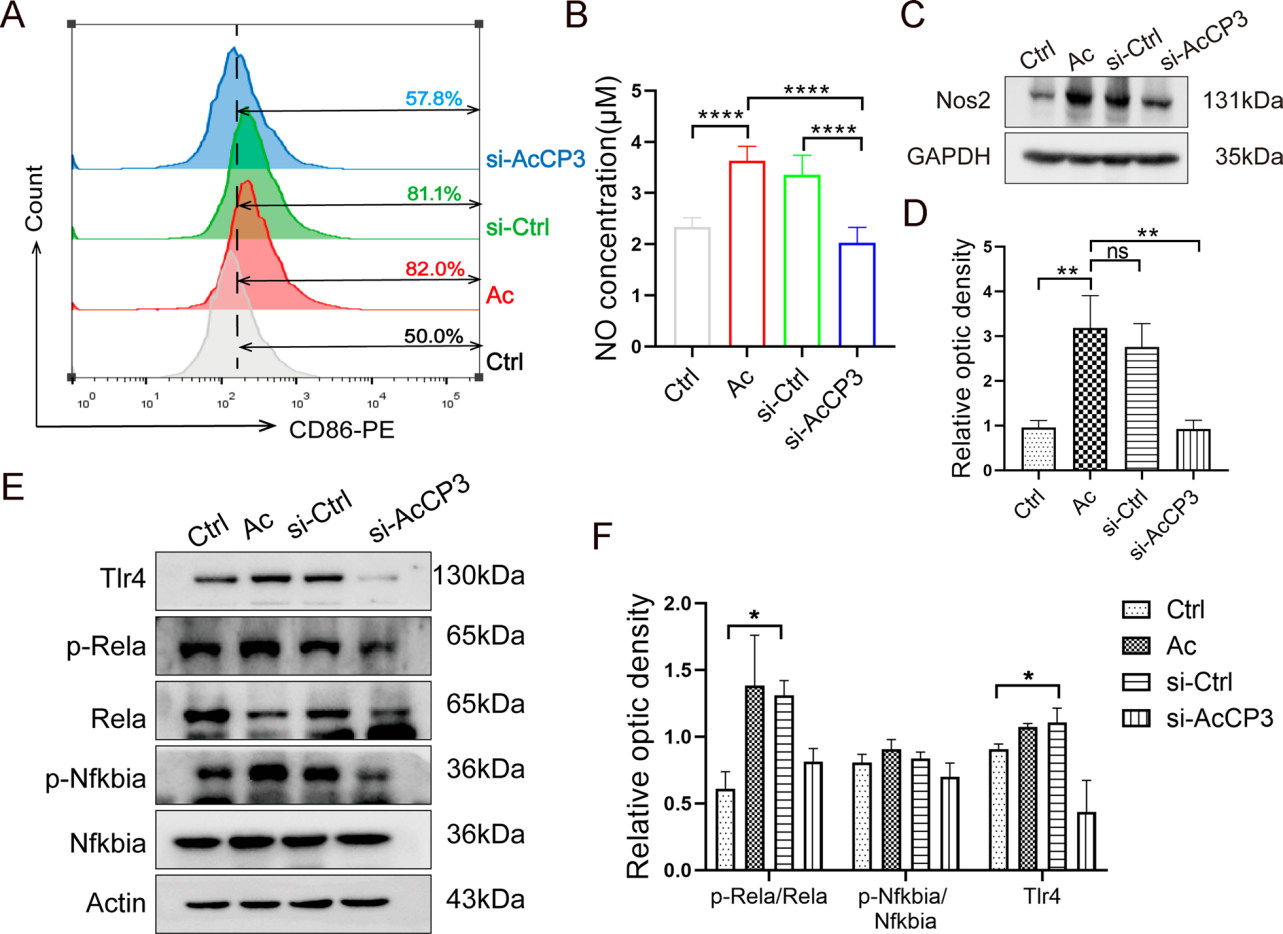
*A. castellanii* cysteine protease 3 (*Ac*CP3) suppresses M1 polarization via TLR4/NF-κB pathway. *Ac*CP3-knockdown, negative control siRNA-transfected, and untransfected trophozoites were co-cultured with macrophages for 12 h. Confluent RAW264.7 cells were used as the control. **A** The Cd86^+^ cell number was analyzed using flow cytometry. **B** The NO content within RAW264.7 cell culture supernatant was examined by Griess assay. **C** The Nos2 level inside RAW264.7 cells was measured using western blotting analysis. **D** The graphs showing the quantification of protein band intensities. **E** The Rela, p-Rela, Nfkbia, p-Nfkbia, and Tlr4 levels were examined using western blotting analysis. **F** The graphs showing the quantification of protein band intensities

**Fig. 7:**
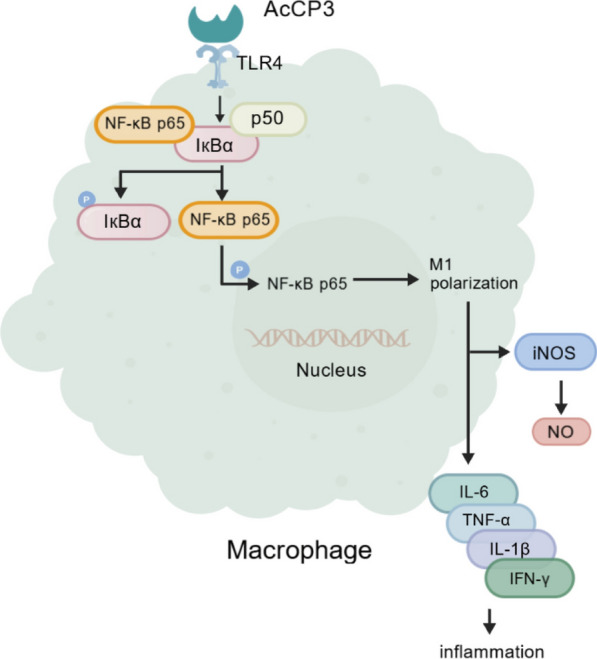
A sketch map showing the mechanism of *A. castellanii* cysteine protease 3 (*Ac*CP3) in promoting M1 macrophage polarization

To further validate that *Ac*CP3 induces M1 polarization in RAW264.7 cells through the TLR4/NF-κB pathway, *Ac*CP3-knockdown *Acanthamoeba* trophozoites were constructed using siRNA and co-cultured with RAW264.7 cells. From Fig. 6A, the Cd86^+^ cell quantity in macrophage/AcCP3-knockdown trophozoite co-culture was lower than that in trophozoite monoculture. *Ac*CP3 knockdown mitigated the *Acanthamoeba* trophozoite-induced upregulation of Nos2 and NO levels of RAW264.7 cells (Fig. 6B–D). From the above findings, *Ac*CP3 knockdown inhibits M1 polarization. The Tlr4, p-Nfkbia, and p-Rela levels were downregulated in RAW264.7 cells co-cultured with *Ac*CP3-knockdown trophozoites (Fig. 6E, F). These findings suggest that *Ac*CP3 knockdown suppressed the TLR4/NF-κB signaling and consequently suppressed M1 polarization in RAW264.7 cells (Fig. 7).

## Discussion

Cathepsin L, a lysosomal cysteine proteolytic enzyme, modulates host immune responses and host–parasite interactions [21]. This study analyzed the function of *Ac*CP3, which belongs to the cathepsin L family, in host–parasite interaction.

CPs are reported to induce inflammatory responses in pathogenic protozoans [22]. For example, CPs from *Entamoeba histolytica* (*Eh*CP1, *Eh*CP2, *Eh*CP5, and *Eh*CP7) induce pro-inflammatory responses of macrophages [23]. This study demonstrated that r*Ac*-CP3 upregulated the levels of Tnfa, Il1b, Ifng, and Il6 and the secretion of Il1b. These findings confirmed that CPs modulate the inflammatory response. *A. castellanii*-secreted proteases are reported to promote Il12 and Il6 production in mouse macrophages by activating protease-activated receptor (PAR) 1 [24]. Additionally, serine protease fibrinogen activator secreted by *Acanthamoeba* trophozoites stimulates IL8 release by activating PAR2 in HCECs [25]. MIp-133, a 133-kDa serine protease, interacts with corneal epithelial membrane phospholipids and promotes arachidonic acid production, pro-inflammatory cytokine release (IL-8, IL-6, IL-1β, IFN-γ, and CXCL2), and the infiltration and apoptosis of polymorphonuclear neutrophils through the activation of the cytosolic phospholipase A2α pathway, contributing to corneal lesion manifestation [26]. Thus, *Acanthamoeba* proteases enhance the production of inflammatory factors in host cells.

The production and secretion of pro-inflammatory factors may be correlated with classically activated M1 macrophages [11, 12]. M1 macrophages participate in initiating and maintaining inflammation and defense responses against pathogens. M1 macrophages express iNOS in which NF-κB was activated. During inflammation, iNOS converts the amino acid l-arginine into NO and l-citrulline. CD86 is a co-stimulatory molecule expressed on M1 macrophages that play a pivotal role in immune responses. M1 macrophages can produce multiple pro-inflammatory cytokines, such as IL-1β, IL-6, IL-8, and TNFα. IL-1β activates the production of IL-8 by binding to IL-1 receptor and inducing the activation of mitogen-activated protein kinase and canonical NF-κB pathways [27]. Autophagy can regulate macrophage polarization. Impaired macrophage autophagy leads to the pro-inflammatory state of M1 macrophages [28]. This study examined the levels of M1 polarization-related factors. Treatment with r*Ac*-CP3 promoted the proliferation of Cd86^+^ macrophages and upregulated Nos2 and NO production. These findings indicate that *Ac*CP3 induces M1 macrophage polarization.

Higher r*Ac*-CP3 concentrations and longer r*Ac*-CP3 durations led to more increased cell viability, likely due to *Ac*CP3’s involvement in inflammation, which promotes macrophage proliferation [11].

NF-κB signal transduction promotes cell survival under physiological conditions, modulates immune reactions in response to external stimuli and inflammation, and regulates homeostasis and metabolism [29]. NF-κB promotes macrophage differentiation toward the M1 phenotype and regulates innate immune responses [29]. The NF-κB proteins comprise NF-κB1 p50/p105, NF-κB2 p52/p100, Rela/p65, Relb and c-Rel. NF-κB targets IκBα within cytoplasm, which along with p100, generates the larger complexes for inhibiting NF-κB. The p65/p50 complex is the predominant component of NF-κB in macrophages. In contrast to other cell types, macrophages exhibit continuous nuclear NF-κB translocation [20].

Furthermore, macrophages express pattern recognition receptors (PRRs) that are responsible for recognizing pathogen-associated molecular patterns (PAMPs) of pathogens. The TLR family members of PRRs recognize molecules, such as microbial LPS, and regulate NF-κB signaling [29]. TLR4 activates the immune system, recognizes trophozoites, and induces the production of cytokines. For example, Tlr4 is upregulated in the brain and lungs of *Acanthamoeba*-infected mice [9]. Similarly, TLR4, IL8, and TNFα are upregulated in HCECs infected with *Acanthamoeba* [30]. This response may be regulated through the NF-κB- and MyD88-dependent pathways [3]. Meanwhile, the main pathways affecting macrophage polarization are the TLR and NF-κB pathways [31]. We hypothesized that *Ac*CP3 serves as an *Acanthamoeba*-associated molecular pattern, interacting with TLR4 on the macrophage surface to activate the NF-κB pathway. Treatment with r*Ac*-CP3 upregulated Tlr4, p-Rela, and p-Nfkbia in RAW264.7 cells. Immunofluorescence analysis demonstrated the increased nuclear translocation of p-Rela. This suggests that *Ac*CP3 activates the cellular TLR4/NF-κB pathway. PDTC pretreatment or *Ac*CP3 knockdown suppressed the r*Ac*-CP3-induced upregulated TLR4/NF-κB pathway-related protein levels and M1 polarization-related factor contents. Thus, the findings of this study demonstrated that *Ac*CP3 promoted M1 macrophage polarization through the TLR4/NF-κB pathway.

CP5 of *Entamoeba histolytica* binds to αvβ3 integrins on the Caco-2 colonic cell surface for inducing NF-κB ubiquitination and inhibitor of κB kinase  activation, triggering NF-κB-mediated pro-inflammatory responses [32]. The CPs of *Blastocystis ratti* upregulate pro-inflammatory factors in mouse macrophages and IL8 production in human colonic epithelial cells through NF-κB [33]. *Leishmania mexicana* cysteine peptidase B modulates host responses through the degradation of NF-κB and its inhibitors (IκBα and Iκβ) and the downregulation of IL12 in infected macrophages to suppress protective Th1 immune responses [22, 23]. CPs belonging to the cathepsin B family of *Naegleria fowleri* promote pro-inflammatory immune responses in BV-2 microglial cells through the NF-κB and AP-1-dependent MAPK pathways [34]. These findings indicate that CPs of protozoan parasites regulate NF-κB-mediated inflammatory responses.

## Conclusions

CP3, a PAMP molecule in *A. castellanii*, interacts with macrophages in the host. *Ac*CP3 enhances M1 macrophage polarization through the TLR4/NF-κB pathway, which may aggravate inflammation through pro-inflammatory cytokine generation. Our results help understand the pathogenesis of *Acanthamoeba* and offer a theoretical framework for preventing and controlling *Acanthamoeba*-related diseases and developing targeted or immunomodulatory treatments for *Acanthamoeba* infections.

## Supplementary Information

Additional file 1: Figure S1 *A. castellanii* cysteine protease 3 (AcCP3) promotes pro-inflammatory cytokine production. Figure S2 Effect of r*Ac*-CP3 protein on RAW264.7 cells viability. Table S1 Primers used for qRT-PCR of *A. castellanii*, HCEC, and RAW264.7 cell genes.

## Data Availability

The raw sequencing data in this study have been deposited at the NCBI under BioProject accession no. PRJNA1298563. Other relevant data generated or analyzed during this study are included in the article.

## Abbreviations

AK: *Acanthamoeba* Keratitis
BSA: Bovine serum albumin
CCK-8: Cell counting kit-8
CPs: Cysteine proteases
DAPI: 4′,6-Diamidino-2-phenylindole
DEGs: Differentially expressed genes
FBS: Fetal bovine serum
HCECs: Human corneal epithelial cells
IFN-γ: Interferon gamma
IL: Interleukin
iNOS: Inducible nitric oxide synthase
KEGG: Kyoto Encyclopedia of Genes and Genomes
LPS: Lipopolysaccharide
MyD88: Myeloid differentiation primary response 88
MOI: Multiplicities of infection
NF-κB: Nuclear factor kappa-light-chain-enhancer of activated B cells
PAMPs: Pathogen-associated molecular patterns
PAR: Protease-activated receptor
PDTC: Pyrrolidinedithiocarbamate ammonium
PRRs: Pattern recognition receptors
r*Ac*-CP3: Recombinant *Acanthamoeba castellanii* cysteine protease 3
RNA: Ribonucleic acid
SDS-PAGE: Sodium dodecyl sulfate polyacrylamide gel electrophoresis
siRNA: Small interfering RNA
STAT1: Signal transducer and activator of transcription 1
TLR: Toll-like receptor
TNFα: Tumor necrosis factor α
