# Supplementary material for: Acanthamoeba castellanii cysteine protease 3 promotes M1 macrophage polarization through the TLR4/NF‑κB pathway
Source: Parasit Vectors. 2025 Oct 29;18:437. doi: 10.1186/s13071-025-07060-y (PMC12570418; doi:10.1186/s13071-025-07060-y)
Supplement: Supplementary file 1 — Additional file 1: Figure S1 A. castellanii cysteine protease 3 (AcCP3) promotes pro-inflammatory cytokine production. Figure S2 Effect of rAc-CP3 protein on RAW264.7 cells viability. Table S1 Primers used for qRT-PCR of A. castellanii, HCEC, and RAW264.7 cell genes. [file 13071_2025_7060_MOESM1_ESM.docx]

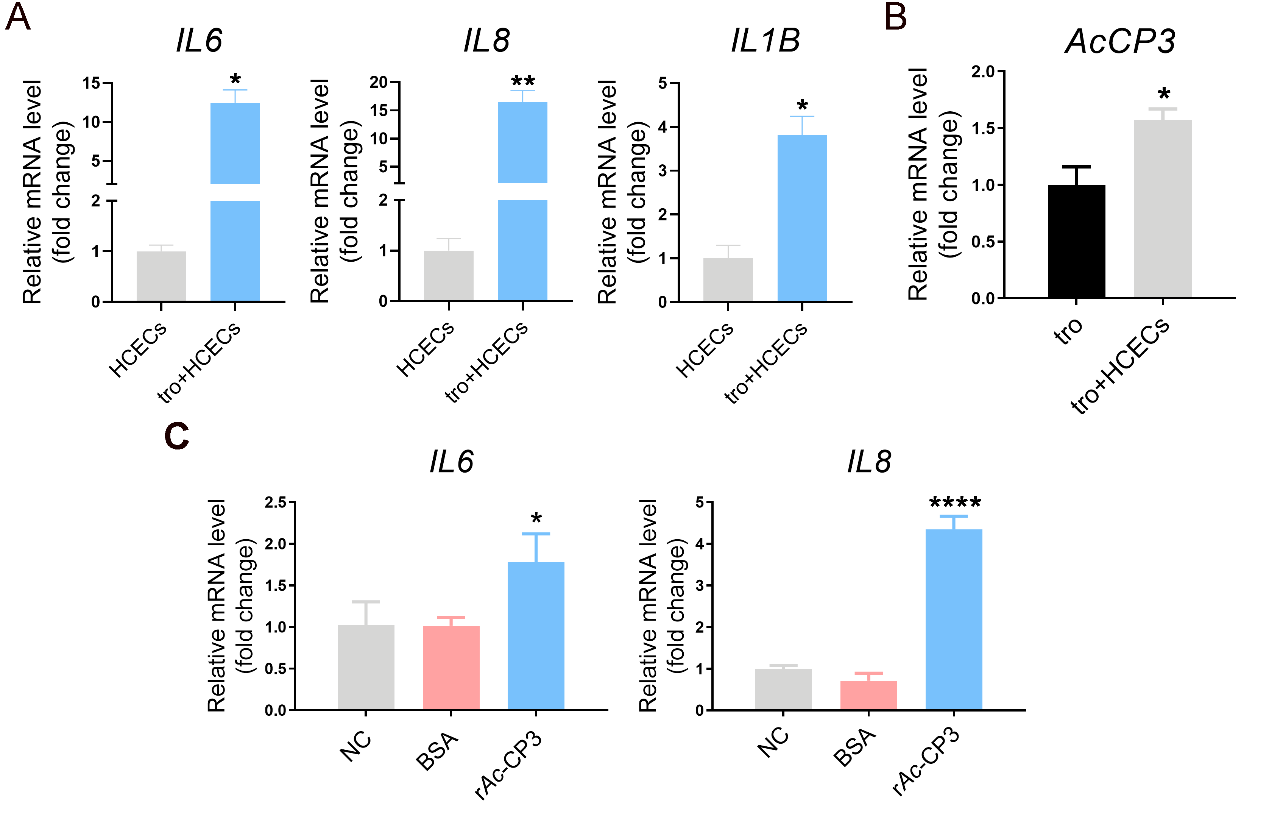
**Figure S1** *A. castellanii* cysteine protease 3 (*Ac*CP3) promotes pro-inflammatory cytokine production.

The mRNA levels of genes encoding inflammatory cytokines within HCECs and *AcCP3* were analyzed by qRT-PCR assay. **A** and **B** *Acanthamoeba* trophozoites were co-cultured with HCECs for 12 h (multiplicity of infection = 1:2; parasite : cells). **C** Cells treated with Tris-HCI (negative control (NC)) and bovine serum albumin (BSA) (non-specific protein) served as the controls. RAW264.7 cells underwent 48 h of recombinant *Ac*-CP3 treatment (20 μg/mL). Means were compared using Student’s t‑test. ^🞲^P < 0.05, ^🞲🞲^P < 0.01 and ^🞲🞲🞲🞲^P < 0.0001*.*


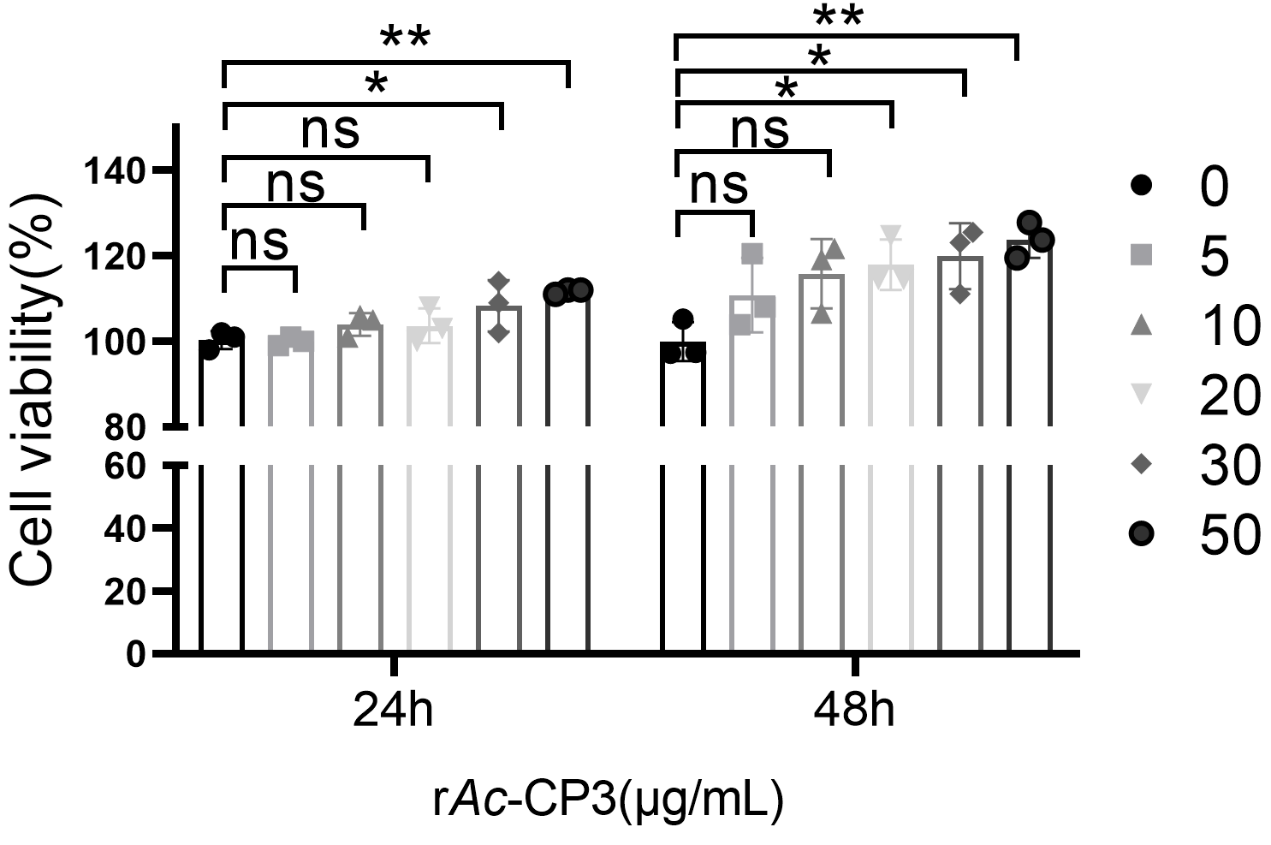


**Figure S2** Effect of recombinant *A. castellanii* cysteine protease 3 (r*Ac*-CP3) on RAW264.7 cell viability.

The RAW264.7 cell viability after 24 and 48 h of r*Ac*-CP3 treatment at varying doses was determined by CCK-8 assay. Student’s t‑test was adopted for mean comparison.

**Table S1 Primers used for qRT-PCR of *A. castellanii*, HCECs and RAW264.7 cells genes.**

| gene | Sense primer(5’-3’) | | | Antisense primer (5’-3’) |
| --- | --- | --- | --- | --- |
| *AcCP3* | CGATGCCTCGCACAACTCCTTC | | | CCACGAGTTCTTGACGAGCCA |
| *Ac-18s rDNA* | TCCAATTTTCTGCCACCGAA | | | ATCATTACCCTAGTCCTCGCGC |
| *Mus-Tnfa* | GTCGTAGCAAACCACCAA | | | GGCAGCCTTGTCCCTTGA |
| *Mus-Il6* | TGCCTTCTTGGGACTGAT | | | TTGCCATTGCACAACTCTTT |
| *Mus-Il1b* | GCTTCAGGCAGGCAGTATCACTC | | | GTGCAGTTGTCTAATGGGAACGT |
| *Mus-Nos2* | TCCTGGAGGAAGTGGGCCGAAG | | | CCTCCACGGGCCCGGTACTC |
| *Mus-Arg1* | CAGAAGAATGGAAGAGTCAG | | | CAGATATGCAGGGAGTCACC |
| *Mus-actin* | TCTACAACGAGCTGCG | | | CAATTTCCCTCTCGGC |
| *Human-IL6* | | GTAGCCGCCCCACACAGACAG | GCCTCTTTGCTGCTTTCACAC | |
| *Human-IL8* | | ACAAACTTTCAGAGACAGCAGA | GTGGAAAGGTTTGGAGTATGTC | |
| *Human-IL1B* | | CCACAGACCTTCCAGGAGAA | GTGCAGTTCAGTGATCGTACAG | |
| *Human-GAPDH* | | TCACCACCATGGAGAAGGC | GCTAAGCAGTTGGTGGTGCA | |
